# Optimizing Carbon Dot—TiO_2_ Nanohybrids for Enhanced Photocatalytic Hydrogen Evolution

**DOI:** 10.3390/ma18051023

**Published:** 2025-02-26

**Authors:** Pinelopi P. Falara, Nikolaos Chatzikonstantinou, Adamantia Zourou, Polychronis Tsipas, Elias Sakellis, Eleni Alexandratou, Nektarios K. Nasikas, Konstantinos V. Kordatos, Maria Antoniadou

**Affiliations:** 1 School of Chemical Engineering, National Technical University of Athens, 9 Iroon Polytechniou St., Zografou, 15780 Athens, Greece; pin.falara@gmail.com (P.P.F.); nick.chatziko@gmail.com (N.C.); adamantia_zourou@outlook.com.gr (A.Z.); 2 Institute of Nanoscience and Nanotechnology, National Center for Scientific Research “Demokritos”, Agia Paraskevi, 15341 Athens, Greece; p.tsipas@inn.demokritos.gr (P.T.); e.sakellis@inn.demokritos.gr (E.S.); nasikas@sse.gr (N.K.N.); 3 School of Electrical and Computer Engineering, National Technical University of Athens, 9 Iroon Polytechniou St., Zografou, 15780 Athens, Greece; ealexan@central.ntua.gr; 4 Department of Military Studies, Division of Mathematics and Engineering Sciences, Hellenic Army Academy, Vari, 16673 Athens, Greece; 5 Department of Chemical Engineering, University of Western Macedonia, 50100 Kozani, Greece

**Keywords:** carbon dots, nanohybrids, photocatalysis, hydrogen production, sacrificial agent

## Abstract

CDs/TiO_2_ nanohybrids were synthesized and tested for photocatalytic H_2_ production from aqueous media through simulated solar light-driven photocatalytic reactions. Firstly, three different types of CDs were prepared through green methods, specifically hydrothermal treatment and microwave irradiation, using citric acid and urea as precursors in varying molar ratios. After a multi-step purification procedure, impurity-free CDs were obtained. The as-synthesized CDs were thoroughly characterized using UV-Vis, FT-IR, and PL spectroscopy, along with HR-TEM. The results revealed that the size and optical and physicochemical properties of CDs can be tailored by selecting the precursors’ ratio and the synthetic approach. The heterostructured CDs/TiO_2_ photocatalysts were formed solvothermally and were analyzed using UV-Vis/DRS, FT-IR, and XPS techniques, which confirmed the effective incorporation of CDs and the improved properties of TiO_2_. The use of sacrificial reagents is among the most common strategies for enhancing H_2_ production from water through photocatalytic processes; herein, ethanol was selected as a green liquid organic hydrogen carrier. A maximum H_2_ production rate of 0.906 μmol H_2_/min was achieved, while the recyclability study demonstrated that the photocatalyst maintained stable performance during multiple cycles of reuse. Thus, optimizing the synthesis conditions of CDs/TiO_2_ nanohybrids resulted in the creation of environmentally friendly and reusable photocatalysts.

## 1. Introduction

Over the past few decades, social awareness regarding environmental and energy matters has grown rapidly. Excessive use of fossil fuels as a primary energy source has led to the discharge of constantly increasing quantities of hazardous gases, such as carbon and nitrogen oxides, into the atmosphere. Meanwhile, the continuous depletion of the available fossil fuels leads to a constantly escalating energy crisis. These essential issues have directed the scientific community towards the pursuit of innovative solutions that combine reduced emissions with the development of renewable fuels [1].

Hydrogen (H_2_) is considered one of the most promising energy sources, causing ever-rising interest from the scientific world. This growing attention is attributed to hydrogen’s remarkable properties, including its high heating value, abundance in nature, versatility in production methods, and environmentally friendly combustion [2]. Among the various hydrogen production methods, the most common is hydrocarbon reforming, which can be performed through steam reforming, partial oxidation, or autothermal processes. However, this method is not renewable, as it relies on the use of hydrocarbons [3,4]. One of the most modern and environmentally friendly methods for hydrogen production is photocatalysis [5,6], a novel technique that utilizes solar energy to produce sustainable green hydrogen. For this purpose, the two main photocatalytic methods are water splitting [7,8] and the reforming of organic compounds [9,10,11]. The photocatalyst in these processes is typically an effective semiconductor exhibiting specific properties such as broad absorption spectrum, high charge separation efficiency, photostability, chemical stability, non-toxicity, low flammability, affordability, and availability [12]. Titanium dioxide (TiO_2_) is the most widely recognized photocatalyst due to its exceptional properties, including suitable energy level configuration, resistance to corrosion, low harmfulness, chemical stability, and cost-effectiveness. However, despite these advantages, TiO_2_ bears notable drawbacks, such as a relatively large energy gap and high electron–hole recombination rate. Thus, the material absorbs only a small fraction of the electromagnetic spectrum (wavelengths below 400 nm) and presents limited potential in practical applications [13,14,15].

On the other hand, carbon nanomaterials (nanotubes, fullerenes, graphene, and carbon dots) are a relatively new and rapidly advancing research area, which have applications across diverse fields such as energy [16,17], environment [18,19,20], and medicine [21,22]. Focusing on carbon dots (CDs), they are composed of a spherical carbon core with various functional groups (carboxyl, carbonyl, and hydroxyl groups) present on their surface [23]. CDs exhibit numerous advantageous properties, including optical characteristics such as electromagnetic radiation absorption and strong photoluminescence, high electronic conductivity, biocompatibility, high quantum efficiency, and an environmentally friendly and straightforward production process. The most commonly employed methods for synthesizing CDs are the hydrothermal and microwave irradiation methods. Both are categorized as bottom-up approaches and are distinguished by their ability to produce small-sized nanoparticles at a low cost [24]. Additionally, these methods allow the adjustment of the CDs’ properties by altering factors such as temperature and precursor concentration. In recent years, the application of CDs as co-catalysts in photocatalysis, in synergy with a semiconductor, has been extensively studied in various technological fields [25,26,27]. CDs are widely used in photocatalytic H_2_ production due to their excellent electron transfer properties, tunable band gaps and ability to facilitate charge separation, reducing electron–hole recombination. Among the most common photocatalysts combined with CDs are TiO_2_, CdS, and graphitic carbon nitride (g-C_3_N_4_) [28]. Specifically, in the synthesis of CDs/TiO_2_ nanohybrid materials, CDs—often doped with heteroatoms such as nitrogen—serve a dual purpose. Firstly, they reduce the energy gap of TiO_2_, thus expanding its absorption spectrum. Secondly, they reduce the thermodynamically favored effect of recombination of charge carriers, as they have the ability of receiving excited electrons, thereby preventing their recombination with holes [29,30].

Therefore, it is proven that integrating carbon nanomaterials can improve the photocatalytic efficiency of TiO_2_ by promoting charge separation and extending light absorption, providing alternatives to noble metal modifications (e.g., Pt, Au, Ag) [31]. CDs are much cheaper than noble metals like Pt, making them a more economical choice for large-scale applications. Simultaneously, carbon nanomaterials are sustainable, whereas Pt and other noble metals are scarce and expensive to extract. Also, CDs are derived from organic sources and do not pose the same environmental concerns as noble metals [32,33].

This study contributes to the advancement of photocatalytic materials, providing a comprehensive comparative analysis of three distinct types of CDs and CDs/TiO_2_ hybrid materials synthesized through green and facile methods, revealing how variations in precursor ratios and synthesis techniques significantly influence their physicochemical properties, ultimately determining their effectiveness in enhancing photocatalytic performance for sustainable hydrogen production. The obtained CD materials were first synthesized via hydrothermal treatment and microwave irradiation, using citric acid and urea as precursors in two different molar ratios, and were fully characterized using ultraviolet-visible (UV-Vis) spectroscopy, Fourier transform infrared (FT-IR) spectroscopy, photoluminescence (PL) spectroscopy, and high-resolution transmission electron microscopy (HR-TEM). In addition, it is important to note that during this work, CDs devoid of impurities were obtained after the purification procedure, which was carried out in consecutive steps. The selection of both the precursors and the synthesis method significantly influences the physicochemical properties of CDs (e.g., surface chemistry, optical properties, etc.). Furthermore, the presence of abundant functional groups on the surface of CDs not only facilitates their easy modification but also enables their combination with various materials, leading to heterostructures with well-defined characteristics. Subsequently, CDs/TiO_2_ nanohybrids were prepared via a solvothermal process, and their optical and structural properties were comprehensively analyzed using various techniques, including X-ray photoelectron spectroscopy (XPS). The resulting hybrid materials were employed as photocatalysts for H_2_ production, utilizing an organic compound as a sacrificial agent. The findings demonstrated that H_2_ evolution is significantly influenced by the nature, chemical structure, and concentration of CDs in the heterostructured photocatalysts. By optimizing the synthesis conditions of CDs/TiO_2_ nanohybrids, environmentally friendly and reusable photocatalysts were developed, presenting enhanced H_2_ evolution rates when ethanol, a representative member of the green liquid organic hydrogen carriers’ family, was selected as the sacrificial agent.

## 2. Materials and Methods

### 2.1. Materials

All chemicals of analytical grade were utilized without further purification. Urea was obtained from Sigma-Aldrich (St. Louis, MO, USA); citric acid was purchased from Honeywell (Charlotte, NC, USA). The nanocrystalline TiO_2_ powder employed in this study was commercial Degussa P25 (Evonik, Essen, Germany). Absolute ethanol (C_2_H_6_O, ≥99%) was provided by Thermo Scientific (Waltham, MA, USA) and Fischer Scientific (Hampton, NH, USA). Deionized water was used during all procedures.

### 2.2. Preparation of CDs and CDs/TiO_2_ Nanohybrids

CDs were synthesized through two distinct methods—(i) hydrothermal treatment process and (ii) microwave irradiation—both of which are described in detail in the following section. The samples named CDs-1 and CDs-2 were prepared by a hydrothermal synthetic route using citric acid and urea in a mass ratio of 1:1 and in a molar ratio of 1:100, respectively. Briefly, the predetermined quantities of citric acid and urea were dissolved into 10.0 mL of deionized water using magnetic stirring to ensure complete dissolution. The resulting solution was then transferred to a Teflon container, which was sealed within a stainless steel autoclave. Subsequently, the autoclave was placed in an oven and heated to 200 °C for 12 h. For the synthesis of the sample labeled as CDs-2-MW, microwave irradiation was employed. More specifically, a molar ratio of citric acid to urea of 1:100 was selected, and the precursors were dissolved in 10.0 mL of deionized water. The solution was vigorously stirred for approximately 10 min and then subjected to microwave irradiation in a standard household microwave of 700 W for 4 min. The resulting product was redissolved in deionized water with the assistance of an ultrasonic bath. In order to remove by-products and non-reactants, some purification methods were employed on all CD samples. Thus, the resulting solution was centrifuged at 6000 rpm for 20 min. The precipitate was discarded; the supernatant was then filtered through standard filter paper and a 0.22 µm pore-sized PET filter. A final purification step was then carried out using dialysis membranes of 0.8–1 kDa retention capability.

For the development of CDs/TiO_2_ nanohybrids, a facile solvothermal method was employed. In particular, certain amounts of the as-synthesized CDs were added to a mixture containing 400 mg of Degussa P25 dispersed in 20.0 mL of deionized water and 6.0 mL of absolute ethanol. The formed mixture was stirred at room temperature for 30 min, transferred to a Teflon-sealed autoclave and heated at 140 °C for 4 h [34]. The resulting photocatalysts were rinsed three times with deionized water, collected by centrifugation and dried at 80 °C overnight.

### 2.3. Characterization Methods

Ultraviolet-visible (UV-Vis) spectroscopy measurements were performed on a Varian Cary 50 UV-Vis spectrophotometer (Agilent Technologies, Santa Clara, CA, USA).

Fourier transform infrared (FT-IR) spectroscopy was carried out with a Jasco FTIR 4200 spectrometer in the range of 400–4000 cm^−1^ using KBr pellets (Jasco, Tokyo, Japan).

Fluorescence spectra of the synthesized CDs were recorded using a Perkin-Elmer (Springfield, IL, USA) LS 45 Luminescence Spectrometer. The slit width was set at 10 nm. The scan speed was adjusted to 480 nm min^−1^.

Structural analysis of the synthesized CDs was performed using high-resolution transmission electron microscopy (HR-TEM). HR-TEM imaging was conducted on an FEI Talos F200i field-emission (scanning) transmission electron microscope (Thermo Fisher Scientific Inc., Waltham, MA, USA) operating at 200 keV, equipped with a windowless energy-dispersive spectroscopy microanalyzer (6T/100 Bruker, Hamburg, Germany). For sample preparation, a drop of CD aqueous dispersion was cast on a holey carbon film supported by a copper grid.

The optical band gap of the prepared heterostructures was measured using UV-Vis diffuse reflectance spectroscopy (UV-Vis/DRS). For this purpose, a Hitachi 3010 spectrophotometer (Hitachi, Tokyo, Japan) was used, fitted with a 60 mm diameter integrating sphere, while BaSO_4_ served as the reference.

X-ray photoelectron spectroscopy (XPS) measurements were performed using a Mg Ka X-ray source with a photon energy of 1253.64 eV, and the spectra were collected with a PHOIBOS 100 (SPECS) hemispherical analyzer. Gaussian–Lorentzian shapes (Voigt functions) were used for the fitting of the recorded spectra after standard Shirley background subtraction.

### 2.4. Photocatalytic Setup for H_2_ Generation and Detection

For the photocatalytic H_2_ generation experiments, a cylindrical Pyrex glass reactor was used, equipped with fittings for the gas inlet–outlet. For the illumination, a 300 W Xenon lamp (Oriel, Stratford, CT, USA) was utilized, and the reactor was positioned 10 cm away. H_2_ detection was conducted using an SRI 8610C gas chromatograph (SRI instruments, Torrance, CA, USA), with Argon (Ar) as the carrier gas. Calibration of the chromatograph signal was performed using a standard mixture containing 0.25% *v*/*v* H_2_ in Ar. The radiation intensity at the location of the reactor was determined at 1 kW/m^2^ (1 sun illumination) using an Oriel Radiant Power Meter, while samples were retrieved every 10 min with an automatic gas sampling valve to monitor H_2_ concentration in the reactor output over the illumination period. For every trial, 100.0 mg of photocatalyst was suspended in 100.0 mL of a 25% *v*/*v* ethanol aqueous solution, as this concentration was found to be optimal for enhancing hydrogen production across various photocatalytic processes [35,36,37]. Throughout the experiments, the solution underwent constant stirring; it was first degassed with Ar flow before switching on the lamp to initiate illumination.

## 3. Results and Discussion

### 3.1. Characterization of CDs

**Figure 1:**
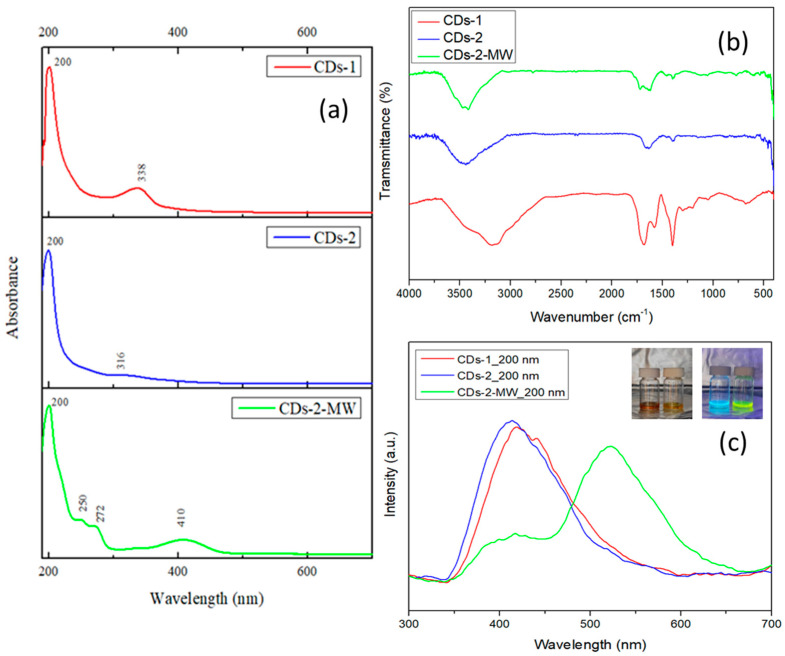
(**a**) UV-Vis absorption spectra of the synthesized CDs; (**b**) FT-IR spectra of the synthesized CDs; (**c**) PL emission spectra of CDs at 200 nm excitation wavelengths (inset: the photograph of CDs-1 (**left**) and CDs-2-MW (**right**) under solar and UV light).

Various techniques were applied to reveal the optical, structural and morphological properties of the as-synthesized CDs. The UV-Vis spectroscopy characterization results of the synthesized CDs are presented in Figure 1a. In the UV-Vis spectrum of the CDs-1 sample, distinct absorption peaks are observed at wavelengths of 200 nm and 338 nm. The peak at 200 nm corresponds to π→π* electronic transitions in carbon–carbon double bonds (C=C), whereas the peak at 338 nm is attributed to n→π* transitions in C=O or C=N bonds. Similarly, in the UV-Vis spectrum of the CDs-2 sample, the first peak appears at 200 nm, consistent with π→π* transitions associated with C=C bonds. The second peak is slightly shifted to a lower wavelength, more specifically at 316 nm, indicating n→π* transitions related to C=O or C=N bonds. However, there is significant differentiation in the UV-Vis spectrum of the CDs-2-MW sample, where new absorption peaks appear. Specifically, although the first peak, observed at 200 nm, remains unchanged, three new peaks emerge at 250 nm, 272 nm, and 410 nm. These peaks are attributed to n→π* transitions in the electrons of the C=N, C=O, and O–H or COOH bonds, respectively. The appearance of these new peaks is possibly due to the use of microwave irradiation as a synthesis method, as they are not present in the CDs-2 dots, which share the same precursor compounds in equal amounts [38,39]. FT-IR spectroscopy was employed to identify the presence of functional groups and chemical bonds in the synthesized materials. The FT-IR spectra of CDs-1, CDs-2, and CDs-2-MW are shown in Figure 1b. More specifically, the peaks located at 1685 cm^−1^ and 1577 cm^−1^ correspond to the stretching vibrations of C=C and C=O, respectively, whereas the peak observed at 1398 cm^−1^ is assigned to the C-O bonds. Additionally, the broad peak, which is present in all samples at approximately 3150–3400 cm^−1^, could be attributed to the stretching vibrations of both O-H and N-H bonds [18,40].

The optical properties of the synthesized CDs were analyzed using photoluminescence (PL) spectroscopy. For each measurement, the excitation wavelength was chosen based on the peaks’ positions observed in the UV-Vis absorption spectra (Figure 1a), corresponding to the maximum absorption bands. The samples were appropriately diluted to prevent any limitations during instrumental measurements. Figure 1c illustrates the emission spectra of the CDs when excited at 200 nm, the wavelength corresponding to their maximum absorption. As observed from the emission spectra, the hydrothermally produced CDs-1 and CDs-2 emit in the blue region of the visible spectrum (380–500 nm). In contrast, CDs-2-MW, synthesized via microwave irradiation, exhibit a weak emission peak in the blue region; however, the sample displays a strong emission in the green region (500–570 nm), extending into the yellow region (575–585 nm). The photoluminescence of CDs-1 and CDs-2-MW is also visualized in the inset of Figure 1c. Furthermore, PL is known to be influenced by the size of the CDs. Yeh et al. [41] clarified this phenomenon, explaining that while particle size does not impact the non-bonding orbital level, significant variations are observed in the π, π*, and σ* orbital levels. This highlights the sensitivity of bonding (π) and anti-bonding (π* and σ*) orbitals to particle size. In this context, the observed redshift in the emission wavelength of CDs-2-MW, when compared to the emission of CDs-1 and CDs-2, is attributed to differences in particle size induced by the synthesis method. Based on the quantum confinement effect, it can be suggested that CDs-2-MW consist of larger nanoparticles than CDs-1 and CDs-2, as smaller particles exhibit higher-energy (shorter-wavelength) emissions.

**Figure 2:**
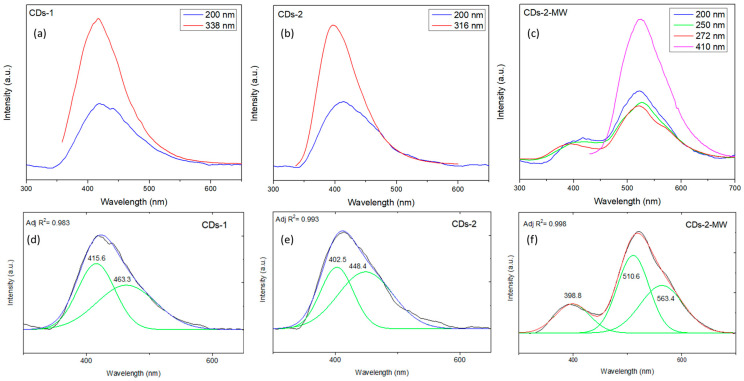
Emission spectra of (**a**) CDs-1; (**b**) CDs-2; and (**c**) CDs-2-MW at different excitation wavelengths, along with the deconvolution of a representative spectrum for (**d**) CDs-1; (**e**) CDs-2; and (**f**) CDs-2-MW.

The emission spectra of CDs-1, CDs-2, and CDs-2-MW, when excited at different wavelengths, are presented in Figure 2. CDs typically exhibit fluorescence properties that are dependent on the excitation wavelength, as commonly reported in the literature. However, there are exceptions where fluorescence emission remains unaffected by excitation wavelength. In this study, the optical behavior of CDs deviated from the usual excitation-dependent fluorescence trend, where the emission peaks gradually redshift with increasing excitation wavelength. Instead, the as-produced CDs demonstrated excitation-independent emission, suggesting that their surface states are uniform and defect-free [42,43]. Additionally, the deconvolution of one representative spectrum for each sample is provided as an inset within the corresponding graph. CDs-1 and CDs-2 exhibit comparable emission peaks, with CDs-1 showing peaks at 415.6 nm and 463.3 nm and CDs-2 displaying peaks at 402.5 nm and 448.4 nm, respectively. On the contrary, CDs-2-MW emitted at 396.8 nm, 510.6 nm, and 563.4 nm.

**Figure 3:**
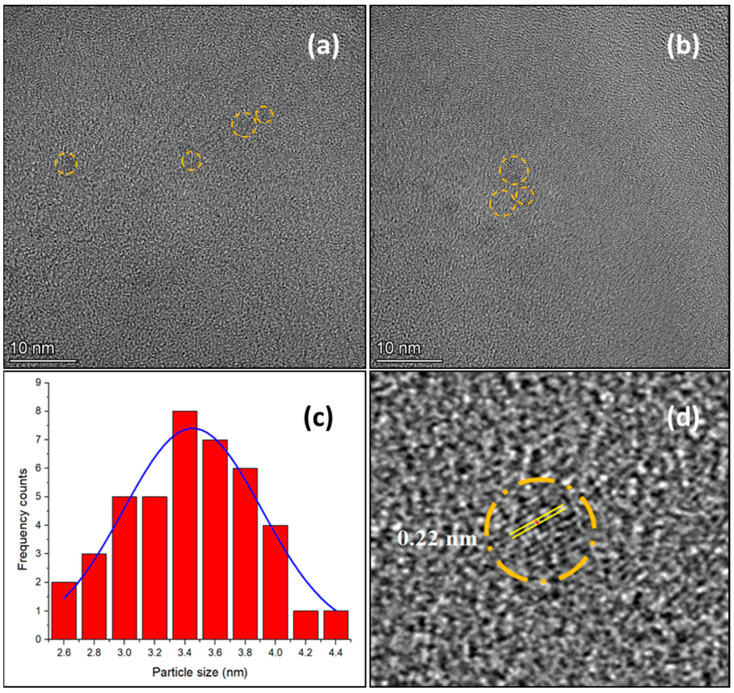
(**a**,**b**) HR-TEM images of CDs-1; (**c**) particle size histogram fitted with Gaussian distribution function; (**d**) graphitic core lattice.

In order to elucidate the morphology and structure of the prepared CDs, HR-TEM measurement was carried out. HR-TEM images of the sample CDs-1 show that the as-synthesized CDs are spherical (Figure 3a,b) with particle sizes ranging from 2.6 nm to 4.5 nm. The Gaussian distribution (Figure 3c) of a sample of 40 particles reveals the mean particle size of 3.45 ± 0.04 nm. In addition, the sample CDs-1 possesses a crystalline structure as indicated by a graphite lattice d-spacing of 0.22 nm (Figure 3d) [44].

**Figure 4:**
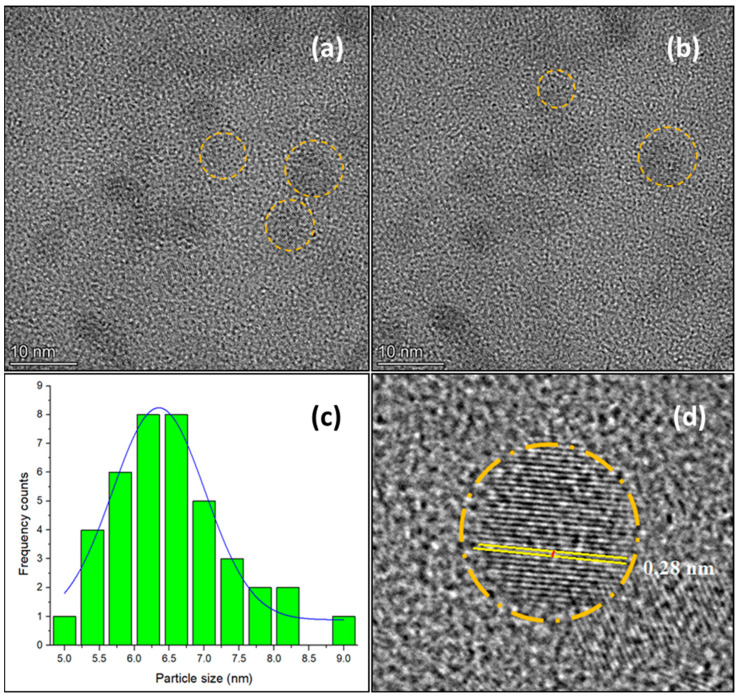
(**a**,**b**) HR-TEM images of CDs-2-MW; (**c**) particle size histogram fitted with Gaussian distribution function; (**d**) graphitic core lattice.

The CDs-2-MW sample was also analyzed using HR-TEM. In Figure 4a,b, the nanoparticles appear spherical, with sizes between 5 and 8.9 nm. A Gaussian distribution (Figure 4c) based on a sample of 40 particles shows an average particle size of 6.35 ± 0.05 nm. Furthermore, the CDs-2-MW sample exhibits a crystalline structure, as evidenced by a graphite lattice d-spacing of 0.28 nm in Figure 4d [45].

The results clearly indicate that the size of the hydrothermally prepared CDs (CDs-1) is nearly half that of the microwave-synthesized CDs (CDs-2-MW). This aligns with the conclusions drawn from PL spectroscopy analysis. In summary, the hydrothermal synthesis produced smaller carbon dots, which correspond to blue light emission, whereas the microwave method yielded larger carbon dots, resulting in a redshift and green light emission.

### 3.2. Characterization of CDs/TiO_2_ Nanohybrids

**Figure 5:**
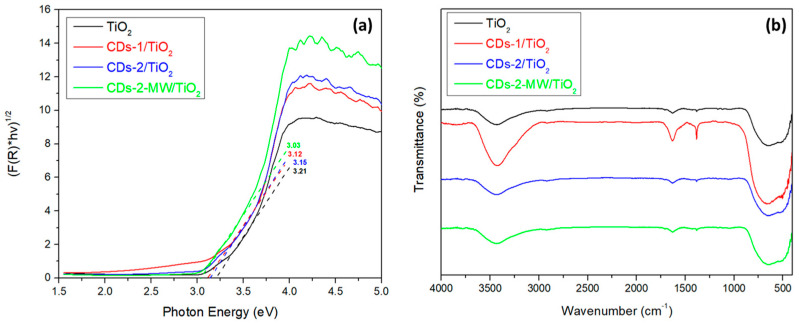
(**a**) Tauc plots illustrating the optical band gaps of pure TiO_2_ and CDs/TiO_2_ nanohybrid samples; (**b**) FT-IR spectra of TiO_2_ and CDs/TiO_2_ heterostructures.

The CDs/TiO_2_ nanohybrid photocatalysts were characterized using UV-Vis/DRS, FT-IR and XPS techniques. In order to study the optical properties of the synthesized nanohybrid materials, UV-Vis/DRS was conducted. The band gaps were calculated using the Kubelka–Munk equation and the Tauc plots (Figure 5a) [46,47,48]. These results indicate great potential, as they demonstrate that the incorporation of CDs enhances photon scattering, thereby improving light-harvesting efficiency. In particular, the calculated band gaps for the synthesized heterostructures are the following: pure TiO_2_ exhibited a band gap of 3.21 eV, while samples CDs-1/TiO_2_, CDs-2/TiO_2_, and CDs-2-MW/TiO_2_ presented band gaps of 3.12, 3.15, and 3.03 eV, respectively. The decrease in the band gap values of the nanohybrids when compared to pure TiO_2_ can be attributed to the chemical interaction between TiO_2_ and CDs, resulting in the formation of Ti–O–C bonds. Consequently, CDs play a crucial role in improving light absorption in the synthesized photocatalyst, as evidenced by previous studies [29,49].

Furthermore, FT-IR spectroscopy was conducted to identify the presence of functional groups and chemical bonds in the synthesized nanohybrids. In the FT-IR spectra of CDs/TiO_2_ heterostructures, which are observed in Figure 5b, the broad peak located at ~660 cm^−1^ is characteristic of the Ti-O-Ti stretching vibrations [37]. As it is noticed, the FT-IR spectra of both TiO_2_ and CDs/TiO_2_ present similar results; this may be attributed to the fact that TiO_2_ is in excess in the heterostructures and peaks attributed to carbon atoms bonds are likely to be obscured. Therefore, further investigation is deemed necessary in order to gain a more comprehensive understanding of the material’s composition and bonding characteristics.

XPS measurements were performed to analyze the chemical state and the surface properties of the synthesized nanohybrids as well as to verify any incorporation of CDs into the TiO_2_.

**Figure 6:**
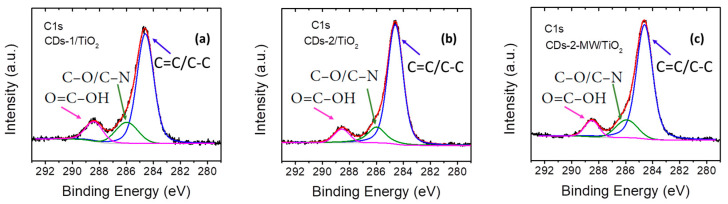
C1s XPS spectra of (**a**) CDs-1/TiO_2_; (**b**) CDs-2/TiO_2_; and (**c**) CDs-2-MW/TiO_2_.

**Figure 7:**
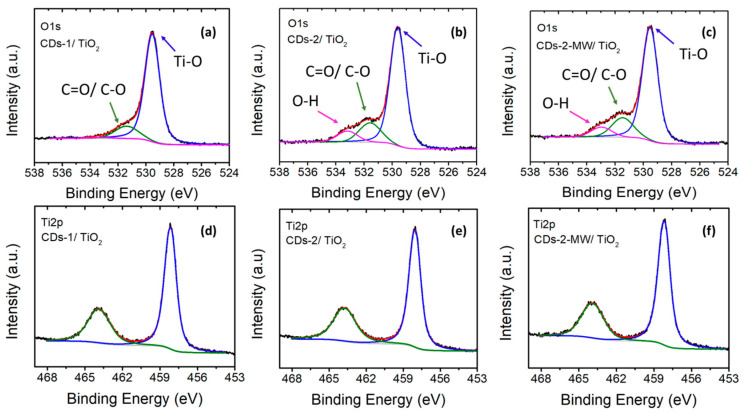
O1s XPS spectra (**a**) CDs-1/TiO_2_; (**b**) CDs-2/TiO_2_; and (**c**) CDs-2-MW/TiO_2_ and Ti2p XPS spectra of (**d**) CDs-1/TiO_2_; (**e**) CDs-2/TiO_2_; and (**f**) CDs-2-MW/TiO_2_.

As shown in Figure 6, the experimental C1s core level peak can be deconvoluted to three fitted peaks. The main peak at ~284.6 eV corresponds to C=C/C–C bonds, whereas the two relatively weak peaks observed at ~286.0 eV and ~288.5 eV correspond to C-O/C-N and HO-C=O functional groups, respectively [44,50]. Regarding the XPS spectrum of O1s (Figure 7a–c), three peaks are observed at binding energies ~529.5, ~531.6, and ~533.0 eV, which can be ascribed to Ti-O, C=O/C-O, and –OH bonds adsorbed on the surface, respectively [51,52,53].

The Ti2p spectra of the three prepared heterostructures (Figure 7d–f) are similar and consist of a doublet with spin-orbit splitting of ~5.7 eV at binding energies 458.2 eV and 463.9 eV, which are attributed to Ti 2p_3/2_ and Ti 2p_1/2_, respectively. These peaks appear at higher binding energies than the corresponding Ti 2p_3/2_ and Ti 2p_1/2_ peaks of pure TiO_2_. This indicates that the linkage between CDs and TiO_2_ is achieved via Ti–O–C bonds [51], demonstrating the well-established concept of CD incorporation into TiO_2_.

### 3.3. Photocatalytic H_2_ Production

**Figure 8:**
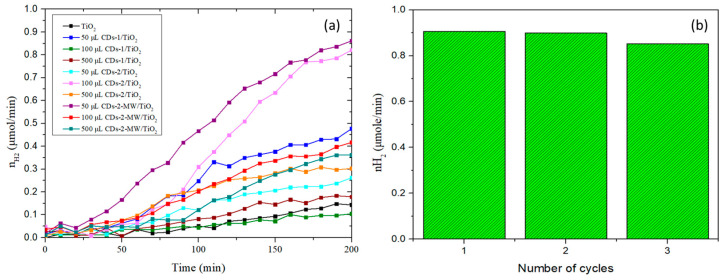
(**a**) Photocatalytic evolution of hydrogen via ethanol reforming in the presence of CDs/TiO_2_ hybrid nanomaterials under 1-sun illumination; (**b**) recyclability tests using the sample 50 μL CDs-2-MW/TiO_2_.

This section presents and discusses the evaluation of the photocatalytic activity. Different volumes (50 µL, 100 µL, and 500 µL, respectively) of the prepared CDs’ dispersion were added to form the corresponding CDs/TiO_2_ heterostructures. The synthesized hybrid nanomaterials were assessed for their photocatalytic hydrogen production ability, using ethanol as a sacrificial agent. For each experiment, 100 mg of the respective photocatalyst was mixed with a 100 mL aqueous solution containing 25% *v*/*v* absolute ethanol. The temporal production of H_2_ in the presence of CDs/TiO_2_ hybrid photocatalysts is demonstrated in Figure 8a.

Regarding the hydrogen production reaction, the diagram indicates that, for most samples, hydrogen production increases over time until it reaches a plateau around 200 min. This suggests that the maximum hydrogen yield is achieved by this point, and further measurements are unlikely to show a significant increase in the rate of the produced hydrogen. The plateau characteristics were influenced by the balance between the amount of photocatalyst and fuel, along with the intensity of the incident radiation. It was observed that, with the exception of one sample (100 μL CDs-1/TiO_2_), all the synthesized nanohybrids produced a greater amount of hydrogen compared to pure TiO_2_. The sample that achieved the highest hydrogen production rate was 50 μL CDs-2-MW/TiO_2_, followed by 100 μL CDs-2/TiO_2_ and 50 μL CDs-1/TiO_2_ in second and third place, respectively. More specifically, the CDs-2-MW/TiO_2_ photocatalyst achieved a rate of 0.906 μmol H_2_∙min^−1^ (543.6 μmol∙h^−1^∙g^−1^), compared to pure TiO_2_, which reached a rate of only 0.150 μmol H_2_∙min^−1^ (90 μmol∙h^−1^∙g^−1^). To ensure that even smaller quantities would not result in a higher H_2_ production rate, 20 μL of CDs-2-MW was also tested for the hybrid photocatalyst. However, the rate was significantly lower, so these results are not included. It is evident that the CDs-2-MW/TiO_2_ heterostructures exhibited superior photocatalytic performance, which aligns with the UV-Vis/DRS results. This hybrid displayed a smaller band gap, leading to significantly improved light-harvesting efficiency.

Additionally, it can be observed that increasing the concentration of CDs in the hybrid nanomaterial does not necessarily lead to a rise in hydrogen production or, consequently, in the photocatalytic activity. This could be because a high concentration of CDs affects light scattering, thereby reducing the amount of light absorbed by the photocatalyst. Therefore, optimizing the nanohybrid does not necessarily require increasing the content of CDs in the hybrid nanomaterial but rather finding the optimal amount that maximizes the broadening of the absorbed wavelength without compromising the photocatalyst’s ability to absorb light. A further evaluation of the hydrogen production results suggests that hybrids containing CDs synthesized using citric acid and urea in a molar ratio of 1:100 (i.e., CDs-2 and CDs-2-MW) exhibited superior photocatalytic activity. This improvement is attributed to the increased nitrogen content, which is claimed to facilitate charge redistribution, thereby enhancing electron transport properties [54]. Lastly, when comparing the two methods for synthesizing CDs, the microwave-assisted approach is considered more effective for nitrogen doping due to its rapid and uniform heating [55]. This characteristic further supports the enhanced photocatalytic performance of the nanohybrid 50 μL CDs-2-MW/TiO_2_. The maximum photocatalytic H_2_ production rates of all prepared nanohybrids are summarized in Table 1.

The stability and recyclability of the CDs/TiO_2_ photocatalysts were also assessed through cycling experiments, as shown in Figure 8b. Specifically, the sample 50 μL CDs-2-MW/TiO_2_ was tested over three consecutive cycles in the presence of ethanol. Each cycle was performed under the same conditions outlined. After each test, the photocatalyst was thoroughly washed with deionized water. The recyclability study demonstrated that the photocatalyst maintained consistent stability during repeated use for H_2_ evolution. These results indicate the potential of CDs/TiO_2_ photocatalysts for practical hydrogen production applications, as their stability over multiple cycles suggests long-term durability. The synthesized heterostructures could be further investigated for integration into photocatalytic systems, such as continuous-flow reactors. Future studies focusing on extended cycling experiments and real operational conditions will be crucial to evaluate their commercial viability.

To emphasize the novelty and advancements of this study in the field of photocatalytic H_2_ production, a review of the recent literature on TiO_2_-based photocatalysts has been carried out. The comparison, as shown in Table 2, aims to illustrate the contribution of this research as well as to distinguish its findings from existing studies.

## 4. Conclusions

Distinct types of CDs were prepared through green methods (hydrothermal treatment and microwave irradiation) using citric acid and urea as precursors in different molar ratios. Following a purification procedure carried out in consecutive steps, CDs devoid of impurities were obtained. The as-synthesized CDs were characterized thoroughly using UV-Vis, FT-IR, and PL spectroscopy, as well as HR-TEM. The results indicated that the physicochemical properties (e.g., size, surface chemistry, optoelectronic properties, etc.) of CDs strongly depend on the precursor’s selection and the synthetic approach. The presence of abundant functional groups on the surface of CDs enabled their combination with TiO_2_ nanoparticles, leading to CDs/TiO_2_ heterostructures with well-defined characteristics prepared via a solvothermal process. The produced CDs/TiO_2_ nanomaterials were thoroughly characterized using UV-Vis/DRS, FT-IR, and XPS techniques, confirming the successful incorporation of CDs and the enhanced properties of TiO_2_. Subsequently, the hybrids were employed as photocatalysts for H_2_ production in aqueous media utilizing ethanol as a sacrificial agent. The obtained results demonstrated that H_2_ evolution is significantly influenced by the nature, chemical structure, and concentration of CDs in the heterostructured photocatalysts. In conclusion, optimizing the synthesis conditions of CDs/TiO_2_ nanohybrids led to the development of environmentally friendly and reusable photocatalysts, achieving enhanced H_2_ evolution rates of up to 0.906 μmol H_2_/min, which may be attributed to the strong interfacial interaction between CDs and TiO_2_, which promotes efficient charge separation, using ethanol as a representative green liquid organic hydrogen carrier.

## Figures and Tables

**Table 1:** Photocatalytic H_2_ evolution rates of all prepared samples.

Sample	Maximum H_2_ Production Rate (μmol/min)	Maximum H_2_ Production Rate (μmol/(h∙g))
TiO_2_	0.150	90.0
50 μL CDs-1/TiO_2_	0.465	279.0
100 μL CDs-1/TiO_2_	0.110	66.0
500 μL CDs-1/TiO_2_	0.179	107.4
50 μL CDs-2/TiO_2_	0.262	157.2
100 μL CDs-2/TiO_2_	0.846	507.6
500 μL CDs-1/TiO_2_	0.315	189.0
50 μL CDs-2-MW/TiO_2_	0.906	543.6
100 μL CDs-2-MW/TiO_2_	0.417	250.2
500 μL CDs-2-MW/TiO_2_	0.378	226.8

**Table 2:** Summary of recent TiO_2_-based photocatalysts for H_2_ evolution.

Photocatalyst	Co-Catalyst	Sacrificial Agent	Maximum H_2_ Production Rate	Ref.
CDs/TiO_2_	-	25% *v*/*v* ethanol	0.906 μmol/min 54.36 μmol/h	This work
			or	
			543.6 μmol/(h∙g)	
	-	15% *v*/*v* methanol	603.92 μmol/(h∙g)	[51]
	-	25% *v*/*v* methanol	9.8 μmol/h	[56]
	- Pt	0.3 M triethanolamine	472 μmol/(h∙g) 1458 μmol/(h∙g)	[57]
CDs/g-C_3_N_4_/TiO_2_	Pt	10% *v*/*v* triethanolamine	580 μmol/(h∙g)	[58]
Graphene/TiO_2_	Pt	25% *v*/*v* methanol	668 μmol/h	[59]
g-C_3_N_4_/TiO_2_	-	20% *v*/*v* methanol	110 μmol/(h∙g)	[60]
Black phosphorus quantum dots/TiO_2_	-	20% *v*/*v* methanol	112 μmol/(h∙g)	[61]
Red phosphorus/TiO_2_	Pt	-	215.5 μmol/(h∙g)	[62]
Au/TiO_2_	-	30% *v*/*v* ethanol	360 μmol/(h∙g)	[63]
Cu/TiO_2_	-	10% *v*/*v* methanol	18 mmol/(h∙g)	[64,65]
NiO/TiO_2_	-	25% *v*/*v* methanol	337 μmol/(h∙g)	[66]

## Data Availability

The original contributions presented in this study are included in the article. Further inquiries can be directed to the corresponding authors.
